# Psychosocial functioning in personality disorders

**DOI:** 10.1186/s40479-025-00326-y

**Published:** 2025-11-12

**Authors:** Ueli Kramer, Chiara De Panfilis, Rasa Barkauskiene, Katja Bertsch, Joost Hutsebaut, Andres Kaera, Mariana Mendoza-Alvarez, Mickey T. Kongerslev, Babette Renneberg, Christian Schmahl, Michaela Swales

**Affiliations:** 1 Department of Psychiatry- CHUV, University Institute of Psychiatry, University of Lausanne, Route de Cery 1, Prilly-Lausanne, CH-1008 Switzerland; 2 University of Parma, Parma, Italy; 3 Vilnius University, Vilnius, Lithuania; 4 University of Würzburg, Würzburg, Germany; 5 De Viersprong National Institute of Personality Disorders, Amsterdam, Netherlands; 6 Kanta-Häme Central Hospital, Hämeenlinna, Finland; 7 University of Antwerp, Antwerp, Belgium; 8 Mental Health Services West, Region Zealand, Denmark; 9 Freie Universität Berlin, Berlin, Germany; 10 Central Institute of Mental Health, Mannheim, Germany; 11 Bangor University, Bangor, UK

**Keywords:** Personality disorders, Psychosocial functioning, Recovery, Social functioning, Interpersonal functioning, Occupational functioning

## Abstract

The present paper takes a broad perspective on the psychosocial functioning in adult patients with personality disorders. We start with a working definition, then we report on psychosocial functioning in personality disorders from both categorical and dimensional perspectives of personality disorder. We add a section on assessment tools which may be used in this context. We then address the question of how problematic psychosocial functioning may be addressed in psychotherapy and other treatment contexts, when it comes to supporting the person’s move towards sustained recovery. We add a lived and living experience perspective to psychosocial functioning and recovery. We end with recommendations for future research in the domain of psychosocial functioning.

## Background

Individuals with personality disorders (PDs) tend to present with a high level of suffering, which may, under certain conditions, extend to their families, partners, close ones and colleagues. PDs may result in high costs related to absenteeism from work, intensive use of health services, ineffective parenting requiring additional clinical and social services, elevated usage of social security disability payments, high costs related to emergency health center usage due to patterns of substance use, aggressiveness and impulsivity, suicidal and self-harming behaviors, as well as may have an impact on overall health and mortality [1–5]. Some of these problems may have legal consequences, increasing society’s financial burden related to PDs [6, 7]. Prevalence of PDs in adults varies between 3% and 9%, and between 20% and 40% in psychiatric settings; around 10% of the medical emergency visits involve problems consistent with severe PDs [8, 9].

The aim of this paper is to summarize what is known about psychosocial functioning in adult patients with PDs. This will be achieved by starting with a working definition and discussing the research from both categorical and dimensional perspectives of PD. The paper will also discuss how problematic psychosocial functioning may be addressed in psychotherapy and other treatment contexts to support sustained recovery. We add a lived and living experience perspective to psychosocial functioning and recovery. The paper closes with conclusions, limitations and recommendations for researchers.

## Main arguments of the paper

### What is psychosocial functioning, and why is it important?

Humans interact with their social environment and the understanding that clinical distress, or a mental disorder, may have an impact on the transaction between these affected humans and their environment is not new [10]. There is a circularity: such transactions impact the self and the self-in-interaction, and the latter may impact the social environment.

Psychosocial functioning comprises the individual’s appropriate functioning in family, intimate and other interpersonal relationships and a healthy adaptation to work, leisure and academic contexts. Thus, psychosocial functioning refers to a person’s ability to carry out social roles and perform activities in daily life, including in social/interpersonal, school/work, recreational and basic (i.e., self-care) functional realms. Appropriateness and health may be marked by mutuality, reciprocity, awareness and empathy. Conversely, impairments in psychosocial functioning in PDs may result from limitations in various mental capacities (e.g., emotional awareness and expression, self-esteem regulation, social cognition, reality testing; [11]. One could argue that deviations from the normative or expected level of psychosocial functioning are already at the core of diagnosing PD and its severity [12], and impairments are evident in social, occupational, and interpersonal functioning and account for the societal and economic impact of PDs [3, 12]. According to qualitative research [13], users and consumers of mental health services tend to emphasise the importance of focussing on the “practical” aspects of living with PDs and the “process” of recovery from PDs.

A possible determinant of psychosocial dysfunction in persons with a PD diagnosis may be their core difficulties in interpersonal relationships, which prevents them from establishing and maintaining supportive social networks [14]. This distinguishing feature has led authors to propose that PDs may represent “interpersonal disorders” [14–16]. These core interpersonal difficulties, under certain conditions, can lead to conflict with other people, which can show up in their social networks and in work environments. Thus, interpersonal dysfunction may affect both social and occupational functioning, which are generally considered the two components of the construct of psychosocial functioning [12, 17–19].

Social functioning refers to an individual’s interactions with their environment and the ability to fulfill their role within such environments as work, social activities, and relationships with partner and family [20]. Occupational functioning has been defined as “the qualities of being suited to serve an occupational purpose efficiently and effectively within the physical, occupational, environmental and psychological demands of a unique work setting” [21]. Consistently, the American Psychological Association Dictionary of Psychology defines “occupational adjustment” as the degree to which an individual’s abilities, interests, and personality are compatible with a particular occupation. Therefore, the definition of occupational adjustment emphasizes the interaction between an individual’s personal characteristics and the objective requirements, conditions, and opportunities associated with the job. As such, occupational functioning is likely to encompass a range of the individual’s abilities, including the ability to pursue work-related goals, to interact and cooperate appropriately with others in the workplace, and to balance productivity and leisure activities satisfactorily (https://dictionary.apa.org/).

Finally, it can be argued that the concept of good psychosocial functioning in mental health is closely related to the concept of “recovery” from a difficulty or an illness. In mental health, recovery is described as “the personal, unique process of changing one’s attitudes, values, feelings, goals, skills and/or roles. It is a way of living a satisfying, hopeful and contributing life even with limitations caused by the illness” [22] (p. 15). Thus, addressing psychosocial dysfunction in PD implies a clear focus on the recovery process.

### How to assess psychosocial functioning in personality disorders

The most important naturalistic longitudinal studies evaluating PD-related functional outcomes (i.e., the Collaborative Longitudinal Personality Disorders Study, CLPS, and the McLean Study for Adult Development, MSAD) used the Global Assessment of Functioning (GAF) Scale score of 61 or higher as a measure of recovery (i.e., [23, 19]). Similarly, the GAF was adopted as the main outcome measure for psychosocial functioning in the most recent Cochrane meta-analysis about the effectiveness of psychotherapy interventions [7]. The GAF (and similar, related, scales) offers the advantage of providing clinicians and researchers with a user-friendly way to measure an individual’s adaptation to the demands of adult life. However, the GAF anchor points represent a mixture of actual symptoms and broader social and occupational difficulties, which could be problematic when the pathways of change in symptoms (i.e., towards remission) and in functioning in terms of psychosocial adaptation (i.e., towards recovery) diverge. Consistently, MSAD operationalized a GAF score of 61 with additional requirements to improve its reliability and meaning: in addition to symptomatic remission, “overall good functioning “ or “recovery” was operationalized as a) being able to work competently and consistently over the past two years (including being a stay-at-home parent or carer), and b) having at least one good relationship with a close friend or spouse/partner; the relationship needs to be characterized by regular contact without elements of abuse (i.e [19, 20]). In the same vein, the CLPS evaluated psychosocial functioning by adding to the GAF the Global Social Adjustment scale, rating functional impairment in various relationships, recreation, employment and satisfaction, without contribution from symptoms, on a 1–5 rating scale, with scores < 3 indicating satisfactory functioning [24].

In the MSAD study, the Revised Borderline Follow-up Interview (BFI-R [25]), was systematically used. It is a semi-structured interview focusing on psychosocial functioning and treatment utilization during the two years prior to the interview. This measure allowed to arrive at the assessment of the criteria outlined above as “overall good functioning” (a and b; [19, 20]).

In addition to these standard measures, there is a great heterogeneity of other assessments. Some studies [24, 26, 27] used the Longitudinal Internal Follow-up Evaluation (LIFE; [28]), an interview assessing the lifetime psychosocial functioning, including occupational and interpersonal functioning. Other studies employed a generic self-report measure of social functioning, the Work and Social Adjustment Scale (WSAS), which is specifically designed to assess functional impairment associated with mental disorders across four domains: work, home management, social and private leisure activities, and close social relations [29]. The WSAS has been found to represent a reliable, unidimensional and gender-invariant measure, sensitive to change and severity of mental distress within PD populations [30].

As for the related construct of interpersonal functioning, the Structured Psychopathological Interview and Rating of the Social Consequences of Psychological Disturbances for Epidemiology (SPIKE; [31]) includes sections evaluating perceived social support, emotional warmth and conflicts within an individual’s social networks and partnership, which have been employed as measures of deficits in interpersonal functioning associated with DSM-IV PD dimensions in a large epidemiological sample [32]. A multisurface interpersonal circumplex (IPC) method, evaluating not only an individual’s problems with relating to others, but also their sensitivities to others’ behaviors can also be used to shed light on both an individual’s difficulties in interpersonal behavior and the perceived impact of others’ behavior on that individual (see [33]). Using this method, research has begun to describe the unique interpersonal problems and sensitivities of people with PD, such as OCPD [34] and grandiose and vulnerable narcissism [35]. In addition, the Structural Analysis for Social Behavior (SASB [15]), can be cited as a relevant method to assess the interpersonal circumplex from a clinically relevant perspective, which is also consistent with dimensional conceptions of PDs [36].

While there is a lack of consensus on a single measure for assessing psychosocial functioning in PD, the present section offers a shortlist of assessment procedures that may prove relevant. The selection of which is the most appropriate depends on contextual determinants, and there is a compelling need for standardized, multidimensional measures across studies to enhance cross-study comparability. For instance, in their review on social and occupational functioning in borderline personality disorder (BPD), Dhar et al. [13] highlight that studies assessing occupational functioning with specific, dedicated measures are indeed lacking. They further suggest that quality of life measures should also be used to identify the different domains that contribute to an individual’s subjective state of suffering. Importantly, given the core difficulties with interpersonal relationships of individuals with a PD diagnosis, measures of quality of life should also include subjective relational aspects, such as satisfaction with self-realization in the areas of work, close relationships, friends, hobbies, sex, health, and family, with the contact with friends, neighbors and family of origin [37]. In this regard, novel methodological approaches such as ecological momentary assessment (EMA) are particularly suited to capture real-time and context-sensitive functioning, such as, for instance, interpersonal functioning during daily interactions with significant others [38].

Incorporating a social-cognitive perspective when investigating psychosocial functioning in PD is crucial in this context. Patients with PD may show unique difficulties in several cognitive processes, such as the perception, judgement and memory of social stimuli, as well as peculiar dispositions, expectations, beliefs or “priors” that in turn shape these social cognitive processes [39–41]. Such biased patterns of social processing are likely to affect their way of relating to others. Flechsenhar et al. [42] proposed investigating not only individual differences in capacities relevant for social functioning and their underlying mechanisms, but also the possibility of (un)learning, modifying, or improving the capacity “to be social”. This social learning framework will allow researchers to investigate dynamic changes in social capacities, which are central to the construct of recovery [42].

Assessment of psychosocial functioning must consider the individual’s ethnic, cultural and social background, as behavioral patterns that might appear dysfunctional in one context may instead reflect adaptive responses in other contexts. Unfortunately, there are marked variations even in the recognition and diagnosis of PD across diverse cultures; at the same time, intrinsic to a PD diagnosis is the concept of a “marked deviation from the expectations of the individual’s culture”. Thus, it is important to employ assessment instruments that are normed to the population being assessed and are culturally sensitive, both for diagnosing PD and evaluating the level of psychosocial functioning [12, 43].

### What we know about psychosocial dysfunction in personality disorders

In this section, we will summarize what we know about psychosocial (dys-)function in PDs, both from a categorical and dimensional perspective, with most research conducted within the former.

Psychosocial dysfunction may be a major determinant of the burden associated with PD, from the perspective of both the mental health practitioners *and* the affected individuals and their carers and/or family members. BPD is associated with particularly high levels of functional impairment, not only compared to other mental disorders, such as anxiety or depressive disorders, but also compared to somatic illnesses such as rheumatoid arthritis, epilepsy, diabetes, or Parkinson’s disease [2, 3, 5, 9]. In BPD, these impairments may have an impact on conditions of disability, such as inability to hold a job or achieve good work or school performance, high direct and indirect medical costs (e.g., sick-leave days and high likelihood of receiving social security disability payments over long time periods), and low probability of being married or living with a partner [9, 44–47].

More broadly, PDs appear to be a more important predictor of poor quality of life and satisfaction with social functioning than sociodemographic variables, somatic disorders, and DSM-IV axis I disorders [37]. Overall, certain patients with PD may find it difficult to achieve and to maintain satisfactory occupational functioning, exhibit high rates (60%) of occupational disability [48], present with low perceived interpersonal support and frequent problems in the intimate, occupational and interpersonal domains (over the past year; [49]). Patients with BPD and Schizotypal PD (SPD) exhibit worse global and occupational functioning than people with other PDs, such as avoidant PD (AvPD) and obsessive-compulsive PD (OCPD; [24, 50]; indeed, in most studies, OCPD is not associated with occupational dysfunction [3, 24, 51]. However, among employed individuals, OCPD symptoms are associated with conflicts in the workplace [51] and with burn-out, occupational stress and high workload and depression, which represent major components of the global burden of disease [52]. As for social and interpersonal functioning, all PD dimensions have been associated with difficulties in these domains, such as having no partner, conflicts and distress with friendships, reduced social networks and low perceived social support [32]. Furthermore, patients with OCPD may exhibit unique interpersonal difficulties, such as the tendency to act in “controlling” ways in interpersonal relations, also discussed as “vindictive” or “cold”, an intolerance to interpersonal warmth and closeness, and a limited capacity to see things from another’s point of view [34].

Prospective longitudinal studies have also pointed out that patients with BPD, Schizotypal PD, Avoidant PD and OCPD exhibit a slower improvement in global psychosocial functioning over time than patients with major depression [24]. In particular, between 50% and 70% of patients with BPD achieved symptomatic remission in the long term but displayed rather small improvements in psychosocial functioning after years of follow-up [20, 24, 53]. These dysfunctions especially concern the relationship domain, although in community samples to a lesser extent than in clinically diagnosed individuals with BPD [54].

A recent systematic review supported the (continual) presence of impairments in social and occupational functioning in individuals with BPD (as well as those in clinical remission) with varying levels of impairment (mild to severe). Furthermore, any improvements in levels of functioning are reported to be at risk of being lost subsequently [13]. Taken together, these results indicate that symptomatic remission is substantially more common than sustained recovery from BPD, and more generally from personality pathology. Fostering the latter remains a major health-economic challenge. Interestingly, most of these studies have focused on the categorical conception of PDs, with a prevalent focus on BPD. While this is consistent with the larger literature at hand, it also reminds us to be cautious when interpreting these results in terms of the dimensional classifications of PDs, in particular the severity criterion of PD. From what is reported above, we can speculate that severe PDs is associated with a stronger impact on all psychosocial functioning variables, but this hypothesis should be tested systematically.

Our focus on adult psychosocial functioning in PDs does not suggest that these questions start with the individual’s entering adult life. It has been suggested that major health and economic benefits may be achieved when the impact of PDs on psychosocial functioning is prevented or reduced as early as possible [55, 56]. This redirects the focus from treatment in a late stage to prevention in an early stage of the disorder and more specifically to adolescence as a critical juncture in the emergence and persistence of psychosocial disability. Impairments in self and interpersonal functioning can interfere with social and academic functioning in adolescence [57–59] and may signal longitudinal risk for pervasive poor functioning during transition to adulthood [60, 61], resulting, for example, in a 22-fold increased risk of unemployment at age 20 for adolescents diagnosed with BPD during adolescence [1].

### What people with lived or living experience have to say about psychosocial functioning and recovery

Focusing on psychosocial functioning in PD is particularly relevant as it is also consistent with the conceptualization of recovery by people with lived or living experience.

To date, the perspectives of PD consumers and their carers have scarcely been investigated in the research literature on psychosocial functioning [62, 63]. Functioning and recovery represent relevant treatment outcomes for people with PD [64]. However, existing qualitative studies point out that consumers and their carers report major concerns about real-life and daily dysfunction in PD. Consumers emphasize the experiential quality of recovery as a “journey” or “progress” or a “process toward stability and well-being” with multiple and multifaceted personal goals, rather than a dichotomous understanding of recovery, encompassing the relationship and occupational sub-domains [14, 62–65]. For instance, individuals with PD tend to report specific and “practical” problems in daily life, such as overwhelming and multi-faceted feelings of loneliness [66]. Furthermore, from the perspective of patients with BPD, personal recovery is represented by practical achievements in the “capacity to work and love”, as indicated by three key themes: (1) love of self and others, (2) contributing through work and study, (3) stability in daily life [67].

Other dimensions of recovery that are relevant for BPD service users include letting go of the past, being involved in meaningful activities, and having healthy relationships [14], as well as developing self-acceptance and self-confidence, gaining control over emotions, improving relationships, employment, and making progress in symptoms like suicidality and self-harm [68]. From the perspective of service users, the main obstacle toward recovery was unstable family relationships, while facilitators of recovery included social support and participation in a specialized therapy program [14].

A survey performed in 2022 by the European Society for the Study of Personality Disorders (ESSPD) among European individuals with lived experience [69] confirmed that the main research priorities from users’ and carers’ perspectives are recovery, (re-integration into society, and meaningful life. Recently, a group of experts by experience (EE) started collaborating with the ESSPD. They believe that the synergy between scientific knowledge and experiential wisdom fosters deeper understanding and more effective problem-solving. They emphasize the need to evaluate dimensions of recovery beyond clinical criteria (professional recovery/employment, social recovery) and in every stage of (psychosocial) life, as well as the involvement of loved ones in recovery. They further pointed out the need to consider the entire range of personality disorders and include groups with special needs when studying psychosocial functioning and recovery in PD [70].

In summary, people with lived and living experience of recovery emphasize the importance of functioning effectively in social and occupational settings. These features should be incorporated into working concepts of personal recovery from PD and represent major treatment targets.

### Treatments supporting recovery in personality disorders

Treatments of problems related to PDs are *psychosocial* in nature [6, 7, 71]. Treatment research mainly focused on BPD and the most recent Cochrane reviews concluded that several empirically supported psychotherapies, but no pharmacological intervention, are effective in decreasing BPD symptoms severity [7, 72]. However, there was only low-quality evidence that psychotherapy may result in small improvement in psychosocial functioning in BPD; furthermore, these improvements did not meet the criterion of minimal clinical relevance and were not sustained over time [7]. Another meta-analysis, though, reported ten psychotherapy studies which assessed interpersonal functioning [73] and showed a small to moderate effect (g = 0.41; 95% CI, 0.09-073), on average, for reduction in problematic interpersonal functioning and in other psychosocial outcomes. These initial findings represent promising results that highlight the need of further studies assessing the impact of psychotherapy on functional outcomes [74].

Importantly, qualitative research indicated a discordance between the goals of individuals with PD diagnoses versus the goals of current psychotherapies. For instance, service users with BPD reported that psychotherapies for BPD often had a focus on specific areas, like self-harming or relationships, but those other goals closer to personal recovery were only indirectly or insufficiently addressed [68].

Finally, while psychotherapy is the treatment of choice for PD, in clinical practice, most patients with severe PD still receive unstructured and unspecific treatment as usual [75–77]. For instance, research has demonstrated that in no country is the number of certified clinicians in empirically supported treatments sufficient to meet the demand posed by BPD treatment seekers [78]. Furthermore, up to half of BPD patients might not respond well to psychotherapy [79]. These factors contribute to an even higher risk of limited effects on psychosocial functioning, including deterioration and disability.

### Addressing the challenges posed by variable psychosocial functioning in the treatment of personality disorders

Researchers have addressed the above mentioned challenges of how to foster sustained functional recovery in severe PDs in three ways: (a) by studying mechanisms of change in psychotherapies which are anchored in the patient’s functional domains which can be targeted by treatments; (b) by studying the impact of add-on targeted rehabilitation counseling programs as a way to support recovery in patients; and (c) by directly targeting self and interpersonal functioning in “generalist” treatment contexts.

#### Identifying and targeting mechanisms of change in psychotherapy

A functional domain is defined as an individually problematic area functionally linked with symptom load. It integrates the severity criterion with the trait components of personality, from a functional context-dependent perspective. For example, by focusing on the functional domain of aggression in patients with BPD, Herpertz et al. [80] showed the effectiveness of a modular group approach to address the problems related to aggression. Neukel et al. [81] showed that symptom reduction due to this modular approach was associated with neurofunctional changes at the level of connectivity between the amygdala and the dorsomedial prefrontal cortex and was related to changes in overt interpersonal aggression, indicating that targeted interventions have specific effects on the psychosocial and neurofunctional levels. Such a conception of targeting functional domains in personality pathology is consistent with the afore-mentioned dimensional definitions [82–84], the Research Domain Criteria [85], research on principles of change in psychotherapy [86–91] and critical events in psychotherapy that may be used from the perspective of mechanisms of change [80, 92–95]. The main potential of targeting functional domains lies in the personalization of the treatment. The assumption is that not all patients will need the full package of an evidence-based treatment, but interventions are tailored to the functional profile of the individual patient. For example, a patient who presents with diffuse identity will benefit from a therapeutic focus on moving towards an integrated sense of self, while another patient who presents with emotion dysregulation benefits from a focus on moving towards an emotional balance, ultimately in both potentially contributing to psychosocial rehabilitation and recovery.

#### Identifying and targeting rehabilitation deficits

Patients with PD could also benefit from interventions that directly address occupational functioning. In youth between 15 and 25 years of age with BPD, the status of vocational disengagement (referred to as Not in Employment, Education or Training – NEET) is cross-sectionally associated with older age, not achieving educational milestones, and problematic alcohol use. Furthermore, NEET status is highly variable at baseline, but the proportion of young people who are NEET at baseline compared with an 18-month follow-up is similar. Predictors of NEET status or changing NEET status over 18 months are not achieving educational milestones, unstable interpersonal relationships and unstable identity. The findings suggest that specific vocational interventions, which also incorporate a focus on interpersonal functioning, emptiness, and identity disturbance, are needed to improve functioning in youth with BPD, especially when educational milestones are not achieved [96]. Inclusion of intensive rehabilitation interventions such as Individual Placement and Support, as add-on modular interventions to existing psychosocial interventions, could also target occupational functioning more specifically [97]. A few pilot studies have employed cognitive rehabilitation to promote everyday functioning in BPD [98] or are evaluating specific programs to improve occupational functioning skills in PD [99]. Furthermore, some treatments explicitly focus on the need to engage in a meaningful activity as both a prerequisite for successful treatment and a treatment goal, envisioning a step-by-step process through which the patient is encouraged to “get a life” (i.e., from being involved in a voluntary job, to a paid part-time and then full-time job, with ongoing attention to finances and housing) [100, 101]. Social rehabilitative interventions may also specifically address key difficulties of BPD, i.e., loneliness and poor social networks. According to people with BPD, increased social connection is often a primary treatment goal and a marker of satisfying recovery, which is scarcely targeted, in explicit ways, by existing psychotherapies. Therefore, interventions like vocational and peer support could be needed to reduce loneliness and to support enduring connection with others [102] with a potential impact on psychosocial functioning.

#### Identifying and targeting impairments in self and interpersonal functioning

A different intervention strategy could be to address the severity of impairments in self and interpersonal functioning (i.e., the DSM-5 Alternative Model for Personality Disorders Criterion A [12]) within generalist treatments where individual psychotherapy is not feasible or not available on a large scale. Using criterion A can also inform the type of process that may be expected in treatment: If a patient is functioning at lower levels of severity, a different therapy process will take place (e.g., involving more spontaneous self-exploration on part of the patient), and a different treatment approach may be warranted (e.g., involving less structural support on part of the therapist). As such, evaluating the level of functioning in the self and interpersonal domain can help delineate hierarchical goals, based on the patient’s current level of functioning, to improve psychosocial functioning in the treatment of patients with PD. Importantly, this focus can be pursued in the context of broader and well-structured generalist treatments for PD [103]. In these cases, treatment programs should consistently aim to enhance personality functioning in the long term by improving self-reflection, expression of emotions and taking a change-oriented stance. This can be accomplished by a careful coordination of interventions across multiple levels (therapist, treatment team, institutions; [104]), which could contribute to not only improved levels of self and interpersonal functioning, but also, and most importantly, to a lasting impact on the patient’s psychosocial functioning.

## Conclusions

### Limitations

The present paper does not represent a formal review of existing research about psychosocial functioning in PDs. However, we propose a consistent perspective on psychosocial functioning in PDs and highlight next steps towards a differentiated understanding of the topic. Based on large-scale studies, we have mostly used medical language to conclude that psychosocial functioning may be impaired in certain clinical presentations of PDs, and to propose ways to identify and target these impairments. In doing so, we acknowledge that the perspectives of users, consumers, and family members may seem to take less center stage. However, we affirm that this perspective on psychosocial functioning is vital in developing a differentiated and complete, researched-backed picture. In particular, qualitative research is scarce, except for the interview-based conclusions from persons with lived and living experience.

### Tentative conclusions and recommendations for future research

In sum, research on psychosocial functioning and recovery in PD has focused largely on samples with BPD, less so on the large array of personality pathology presentations. With the advent of the dimensional conceptions of PDs, we recommend studying various PD presentations with regard to their potential impact at the level of psychosocial functioning.

Research generally lacks a comprehensive, flexible, function-related, and specific measure of psychosocial functioning for PD, encompassing the social, occupational and interpersonal domains. There is also a compelling need to employ cross-culturally validated measures and to consider multiple sources of information (patients, family members, peers, clinicians). When focusing on recovery, it is important to include consumers’ driven subjective perceptions of psychosocial functioning and quality of life. Clinicians should also collaboratively work with service users towards identifying treatment targets that are subjectively important to service users, their priorities, and long-term plans on how their targets might be met and which services might be involved [68].

A comprehensive definition of psychosocial functioning in PD should consider the intra-individual variability of the level of functioning among individuals suffering from PD. This perspective includes both the conceptualization of difficulties that arise in response to specific situations and challenges, and also the strengths and abilities that patients can deploy to achieve satisfactory levels of social, interpersonal and occupational functioning. Future studies using validated and agreed-on measures to evaluate social, occupational and interpersonal functioning, from multiple sources, could illuminate the specific patterns of functioning of diverse PD presentations in these various domains. Newer developments in the fields of in-situ self-report and physiological assessments of socio-cognitive functioning, as part of functional domains related to personality pathology, will push the boundaries of situation-bound knowledge of psychosocial functioning and capture real-time, context-sensitive variations.

Regarding treatments to promote recovery, psychotherapy is the first-line treatment for difficulties associated with PDs, but its impact on recovery remains understudied. Yet, targeting psychosocial difficulties, by considering a functional domain perspective to personality pathology, or by directly adding specific modules on vocational counseling to the treatment plan, is the key element in fostering sustained functional recovery in PDs. Psychotherapy research should select psychosocial measures as outcomes in the demonstration of effectiveness. Such research may identify predictors and in-session processes associated with recovery in the domain of PDs. Among these, emotional awareness, experience and expression [91, 105] may be relevant candidates, along with in-session socio-cognitive and interaction competencies ( [106]; see the review [93]). Empirically supported psychotherapy might not always be available on a large scale [78]. However, structured generalist treatments [101, 104, 107] may be scalable and may also help improving psychosocial functioning in PD [108]. This aspect represents the foundation of the active therapeutic stance shared by all the effective therapies for PD: patients with PD experience difficulties that can at times impair their levels of functioning, but researchers and the public need to understand that they are not “disabled”: these limitations do not prevent them from changing their behavior over time [100, 107, 109]. Importantly, this also aligns with the previously described concept of recovery among PD services users as a dynamic “learning process” or “journey” toward accomplishing practical every day, meaningful goals.

## Data Availability

No datasets were generated or analysed during the current study.
